# Adaptive Fuzzy Modal Matching of Capacitive Micromachined Gyro Electrostatic Controlling

**DOI:** 10.3390/s23177422

**Published:** 2023-08-25

**Authors:** Li Cheng, Ruimin Liu, Shumin Guo, Gaofeng Zheng, Yifang Liu

**Affiliations:** 1 Department of Aeronautical and Aviation Engineering, Hong Kong Polytechnic University, Hong Kong; 22043547g@connect.polyu.hk; 2 Pen-Tung Sah Institute of Micro-Nano Science and Technology, Xiamen University, Xiamen 361102, Chinazheng_gf@xmu.edu.cn (G.Z.); 3 School of Mathematical Sciences, Xiamen University, Xiamen 361002, China; shumin_guo@xmu.edu.cn

**Keywords:** capacitive micro-machined gyro, electrostatic control, modal matching, numerical simulation, feedback control

## Abstract

A fuzzy PI controller was utilized to realize the modal matching between a driving and detecting model. A simulation model was built to study electrostatic decoupling controlling technology. The simulation results show that the modal matching can be gained by the fuzzy PI controller. The frequency difference between the driving mode and the detection mode is less than 1 Hz, and the offset of the input DC is smaller than 0.6 V. The optimal proportionality factor and integral coefficient are 1.5 and 20, respectively. The fuzzy PI controlling technology provides a good way for the parameter optimization to gain modal matching of micro gyro, via which the detecting accuracy and stability can be improved greatly.

## 1. Introduction

Micro-electromechanical system technology (MEMS) is an important technology for microsystem integration and has been applied in various fields, such as high-precision sensing [1,2], automobile [3], electronic information [2,4], biomedical [5], aerospace [6], and so on. There are also some novel methods that have been invented to improve the sensing performance of gyros. Mao et al. [7] studied the mode-matching and Sagnac effect in a millimeter-scale-wedged resonator gyroscope. Xue et al. [8] invented the all-polymer monolithic resonant-integrated optical gyroscope. Wang et al. [9] utilized a resonator with spun fiber to suppress the Kerr-effect-induced error in resonant fiber optic gyros. Feng et al. [10] employed a Si_3_N_4_ resonator to improve the long-term temperature bias stability of integrated optical gyroscopes.

The capacitive micro gyro detecting angular velocity based on the Coriolis Effect has become the most widely used angular velocity micro-sensor device with the advantages of low manufacturing cost, good reliability, and easy integration [11,12,13]. However, the resonant frequency of the gyro detection mode will drift because of process errors and ambient temperature. So, the modal frequency between the driving mode and the detection mode should be matched, which is the key that defines the sensitivity and stability of micro-mechanical gyros [14,15].

Presently, there are several modal matching controlling methods, including the quality adjustment method, material characteristic adjustment method, and stress adjustment method [16]. The quality adjustment method that was designed based on theoretical analysis and process experience changes the resonant frequency by changing the resonator quality or mass compensation [17]. The quality adjustment method is easily disturbed by other factors, which limits the overall performance improvement of the gyro. The material property adjustment method changes the resonant frequency by depositing different materials on the surface of the resonator [18,19]. The materials deposition process was complex, and it was difficult to gain the suitable thickness and quality of the material deposition layer; therefore, the matching performance could not be realized easily. The methods of quality adjustment and the material property adjustment are offline control methods and are not suitable for long-term stable control. The above methods need to be implemented during the design and manufacturing process; therefore, they are difficult to achieve and expensive. The stress adjustment method is an online control method and has become the most commonly used method, which does not require implementation during the design and manufacturing process of micro gyros [20,21].

The stress adjustment methods are divided into the temperature adjustment method and the external force adjustment method. The temperature adjustment method changes the resonant frequency by heating the micro-vibration structure locally [22], which has the disadvantages of poor control ability and high power. It is difficult to apply to different material structures due to the different temperature-sensitive characteristics. The external force control method changes the resonant frequency using residual internal stress [23], electric field force, and magnetic field force [24]. Compared with other control technologies, the external force adjustment method is an online control technology, which is beneficial to the sensitivity and stability of the micro-mechanical gyro system with its high flexibility and strong universality [25].

The static control method changes the resonant frequency by introducing an electrostatic force on the harmonic oscillator to achieve modal matching control, and it has become the method with the most development potential in the external force adjustment method [26]. There is a difference between the resonant frequencies of the two directions because of the interference of the manufacturing process and the environment. The feedback control system can be built based on the resonant frequency difference between the two directions. The system adjusts the DC offset acting on the capacitor resonator to change the resonant frequency to achieve modal matching to eliminate resonant errors. Static control is an easily achieved online control method, which is helpful in speeding up the response to noise interference and improving detection accuracy. Electrostatic control technology has become the optimal choice for modal matching control of micro gyro systems [27].

In this paper, we analyze the theoretical derivation in the process of electrostatic regulation of micro-capacitive gyros and build the modal matching system. We build the numerical simulation model based on the Simulink module of MATLAB software (R2018a) to achieve the modal matching of gyro and analyze the controller parameters. The influence of the quality factor on the control process is researched. And the signal evolution law of the modal matching process of the capacitive micro gyro is simulated.

## 2. Analysis of Electrostatic Control

**Figure 1:**
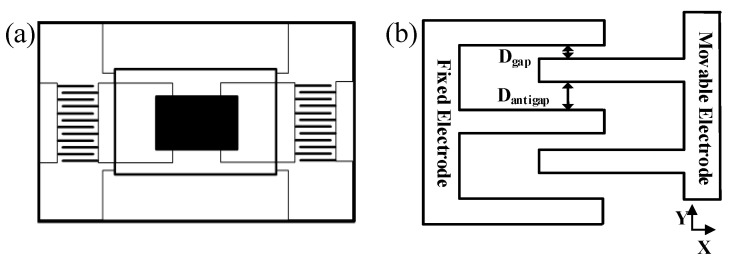
The structural features of capacitive micro gyro: (**a**) the structure of capacitive micro gyro; (**b**) the comb-tooth capacitance structure.

It is shown in Figure 1a that the capacitive micro-mechanical gyro is mainly composed of an inertial mass, internal and external frames, elastic elements, and a comb-tooth capacitor structure. The inertial mass has two degrees of freedom, and the Coriolis force is transmitted to the comb capacitor structure through the elastic element when the gyro vibrates. It is the particularity of the comb capacitor structure that makes both the driving mode (vibration in the X direction) and detection mode (vibration in the Y direction) have only one degree of freedom. The comb-tooth capacitor structure is shown in Figure 1b, which includes a fixed plate and a movable plate. A sinusoidal signal is added to the fixed plate, and a DC bias signal is added to the movable plate to drive the gyro. When the gyro is in resonance, the DC signal and high-frequency signal are filtered out by the band-pass filtering function of the structure of the internal phase-locked loop. The equations of driving can be simplified to:

(1) $$F_{x}=\frac{\partial E}{\partial x}=\frac{\partial C}{\partial x}VA\sin(\omega t)$$

where *E* is the electric field strength between the capacitor plates, *C* is the capacitance value, *V* is the DC bias voltage value applied to the capacitor plates, and *A* is the amplitude of the sinusoidal signal.

While applying the voltage to the comb capacitor structure, there is a slight force in the detection direction due to the particularity of its structure. We can obtain the detection mode electrostatic negative stiffness equation as follows:

(2) $$k_{e}=\frac{\partial F}{\partial y}=-\frac{1}{2}N\varepsilon HL\frac{D_{antigap}^{3}-D_{gap}^{3}}{(D_{antigap}\cdot D_{gap}{)}^{3}}V^{2}=KV^{2}$$

where *N* is the number of comb-tooth capacitor groups. Here, ε, *H* and *L* are the parameters of the capacitor plate of dielectric constant, thickness, and length. Then, *D_antigap_* and *D_gap_* are the structural parameters of the comb-tooth capacitor in Figure 1b, and *V* is the DC bias voltage applied by the capacitor plate. Denote the product of the above parameters and coefficients with *K*. The design of the mechanical structure eliminates the modal coupling between the driving direction and the detection direction. However, there are orthogonal errors between the two resonance directions due to the differences in the manufacturing process [16]. In this paper, we build a modal matching closed-loop control system based on the feedback of the phase between the quadrature error signal and the driving signal. The system eliminates static errors and improves detection accuracy by the PI controller.

When the micro-mechanical gyro vibrates, both the driving mode and the detection mode can be equivalent to a second-order spring damping system, from which the resonant angle frequency of the detection mode can be calculated:

(3) $$\omega_{s}=\sqrt{\frac{k_{eff}}{m_{s}}}=\sqrt{\frac{k_{m}+KV^{2}}{m_{s}}}$$

where *k*_eff_ is the effective static stiffness of the detection mode, *m_s_* is the equivalent mass of the detection mode, and km is the elastic coefficient of the spring damping system equivalent to the detection mode. A closed-loop control system of modal matching based on the characteristic relationship between the input DC bias voltage and the detection terminal signal is built in Equation (3). By adjusting the magnitude of the input DC offset, the resonant frequency between the driving mode and detection mode can be matched, realizing the modal matching of the system stably.

## 3. Construction of the Modal Matching System

**Figure 2:**
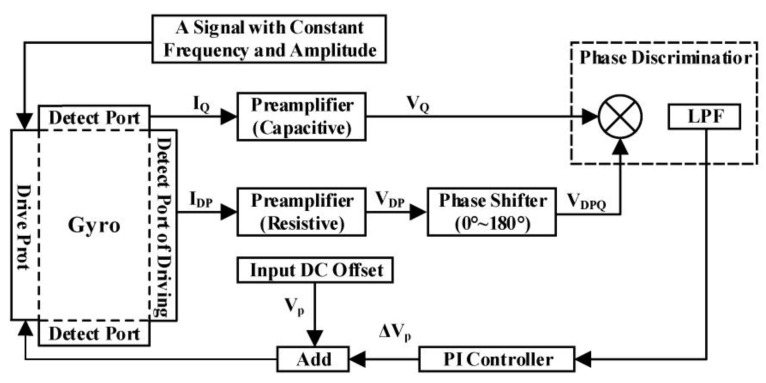
Block diagram of the modal matching control system.

Then, a matching controlling system with voltage has been built to study the modal matching controlling technology with a fuzzy PI controller. The block diagram of the modal matching control system is shown in Figure 2.

The gyro is driven by the constant amplitude and constant frequency signal and the lower DC bias signal. And the signal of the drive module and the detection module of the gyro are amplified through the front transimpedance amplifier and trans-capacity amplifier, respectively. According to the principle of synchronous demodulation, the demodulated signal is the strongest when the two signals are in the same phase as each other [17]. Therefore, we add a phase shift module before signal demodulation to shift the phase 90° according to theoretical calculations. The phase difference of 90° is a good way to make sure the driving modal is perpendicular to the detecting modal and achieve an accurate detection. The signals reach the phase detector, which consists of a multiplier and a low-pass filter, and finally input the PI controller:

(4) $$V_{error}(t)=-\frac{m_{d}R_{f}C_{x}C_{y}V^{2}A_{x}^{2}A_{y}\omega_{x}^{3}\sin\theta }{C_{m}d^{2}}\cos(\delta_{2})$$

where *x* is the coefficient of the driving mode, y is the coefficient of the detection mode, *R*_f_ is the value of the transimpedance amplifier, sin⁡θ is the sinusoidal value of the quadrature error angle, *d* is the parameters of the capacitive plate. We can further calculate:

(5) $$\delta_{2}=\arctan\frac{c_{y}\omega_{x}}{k_{y}-m_{s}\omega_{x}^{2}}=\arctan\frac{2\zeta (\omega_{x}/\omega_{s}{)}^{2}}{1-\frac{\omega_{x}^{2}}{\omega_{s}^{2}}}$$

when two angular frequencies of both the driving modal and the detection modal are equal, the modal matching is realized, and it is calculated according to the above formula:

(6) $$\delta_{2}=\frac{\pi }{2}$$

The modal matching is realized when *V_error_*(*t*) is zero. The PI controller outputs a DC offset signal, which is superimposed on the initial input DC offset signal and then used as the input of the DC offset signal of the gyro. When it is detected that the input of the PI controller is zero, the system reaches modal matching, and the phase difference between the signal of the driving modal and the detected modal is 90°. The modal matching system adjusts the amplitude of the input DC offset signal and uses PI control to adjust the DC offset signal within 0.6 V accurately.

## 4. Simulation and Result Analysis

**Figure 3:**
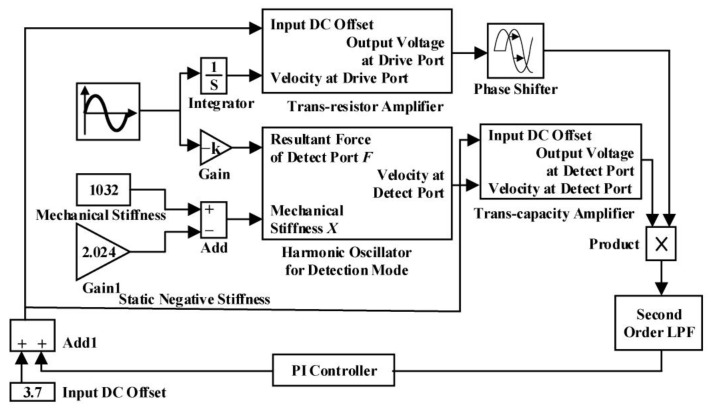
Simulation model of modal matching system.

As shown in Figure 3, the simulation model is built in MATLAB Simulink based on the dynamic characteristics of micro gyro.

The system response characteristics of modal matching and the action rules of various parameters are analyzed via simulation. The system realizes the control without static error through the PI adjustment method, which improves the control precision of the micro gyro.

### 4.1. Analysis of Modal Matching Signal

**Figure 4:**
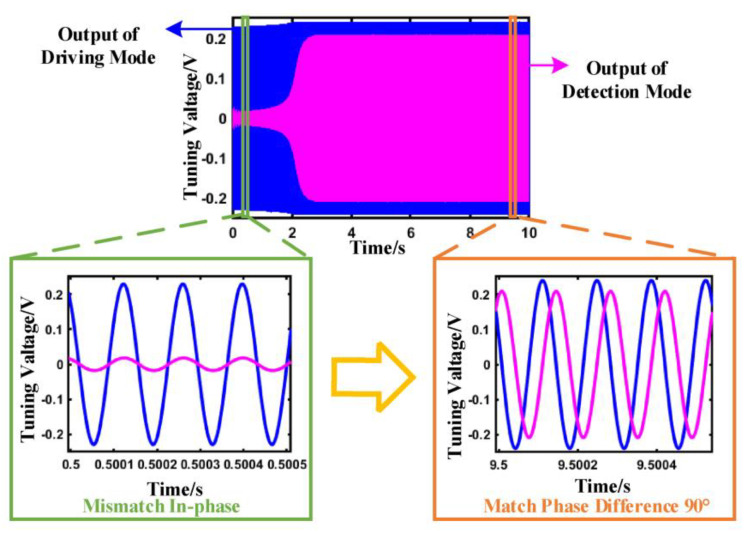
Signal evolution process of system modal matching.

The system simulation model optimizes the characteristics of signal transmission and realizes the signal matching between the two modes through closed-loop control. The output signals of driving and detecting in modal matching are shown in Figure 4, respectively.

The detection signal is weak and has large fluctuations when the micro gyro is not modal matching. When the system realizes the mode matching, the signal of the detection mode of the system gradually increases and tends to be stable through the adjustment of the closed-loop control. There is a phase shift circuit in the control system, which can perform phase shift calculation on the driving signal and improve the sensitivity and sensing accuracy of the signal identification. When the system realizes modal matching, the phase difference of the two signals is 90°.

The signals of the two modes before and after the mode matching are shown in Figure 4. At the time of start-up, the system is in a state of modal mismatch, and the phase difference between the signal of the driving modal and the detection modal is not 90°. The system gradually realizes modal matching via closed-loop adjustment, in which the phase difference of the two signals is equal to 90°.

The output of the PI controller is not an ideal DC signal but a signal with a weak sinusoidal ripple. The driving mode and the detection mode of the gyro are not ideal for mode matching. There will be a frequency difference between them, so the designed system controls the difference within 1 Hz. The amplitude range of the control sine ripple can be calculated as follows:

(7) $$\omega_{d}-\omega_{s}<2\pi \Delta f$$

(8) $$\omega_{d}=\sqrt{\frac{k_{y}-KV^{2}}{m_{s}}}$$

(9) $$\omega_{s}=\sqrt{\frac{k_{y}-K(V+\Delta V{)}^{2}}{m_{s}}}$$

The amplitude range of the sine ripple is ΔV<0.0041 V. We optimize the parameters of the PI controller to meet the system design accuracy.

### 4.2. Optimization and Analysis of PI Control

The influence of the proportional and integral parameters of the PI controller on the modal matching process is analyzed. Under the premise of satisfying the design accuracy, the parameters of the PI controller, which decides the range of the amplitude adjustment of the system, are determined. The impact of the initial input DC offset on the entire system is analyzed finally.

**Figure 5:**
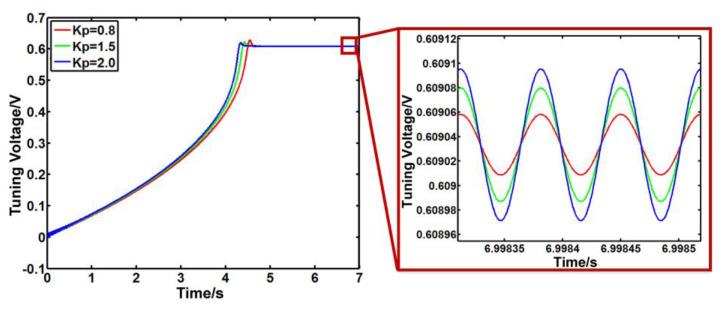
The output signal of PI controller with different *K*_p_.

It is shown in Figure 5 that *K*_i_ is 20, and *K*_p_ is 0.8, 1.5 and 2.0. Here, *K*_i_ is the integral control coefficient, and *K*_p_ is the proportional control coefficient for the PI controller, respectively. As *K*_p_ increases, the stability time of the system decreases, the response speed increases, and the overshoot of the system decreases. However, when *K*_p_ is 2.0, the system oscillates greatly, which means that the excessive *K*_p_ will cause the system to oscillate, resulting in system instability.

**Figure 6:**
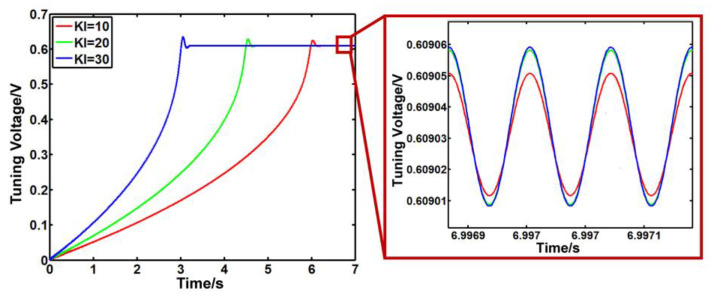
The output signal of the PI controller with different Ki parameters.

As shown in Figure 6, as *K*_i_ increases, the stability time of the system gradually decreases, but the overshoot of the system increases too quickly. Increasing the value of *K*_i_ as much as possible can reduce the stability time.

According to the simulation results, the optimal PI controller parameters are 1.5 for *K*_p_ and 20 for *K*_i_. At this point, the system can achieve a 0.6 V difference adjustment.

**Figure 7:**
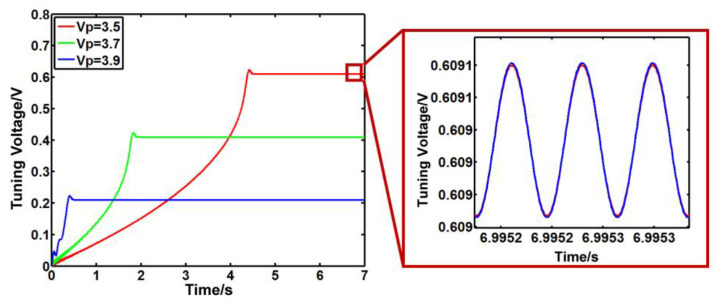
The output signal of the PI controller with different DC bias voltages.

Next, the impact of the input DC bias voltage on the system is analyzed. Take the input DC offset voltage as 3.5 V, 3.7 V, and 3.9 V. As shown in Figure 7, the tunable voltage of the system decreases, and the speed of system response is greatly accelerated with the increase in DC offset voltage. However, the system oscillates strongly before the stability stage with modal matching. The simulation results show that the system can achieve an exact stable stage when the input DC offset voltage is 3.7 V.

### 4.3. Analysis of the Performance of the Control System

The quality factor of capacitive micro-mechanical gyro will be reduced due to the influence of process and environment. According to Equation (10), we simulate the changes in the parameters of the gyro to analyze the change in the quality factor.

(10) $$m\ddot{y}=F_{q}\cos\omega t-c\dot{y}-ky$$

(11) $$c=\frac{\sqrt{k_{s}m_{s}}}{Q}$$

**Figure 8:**
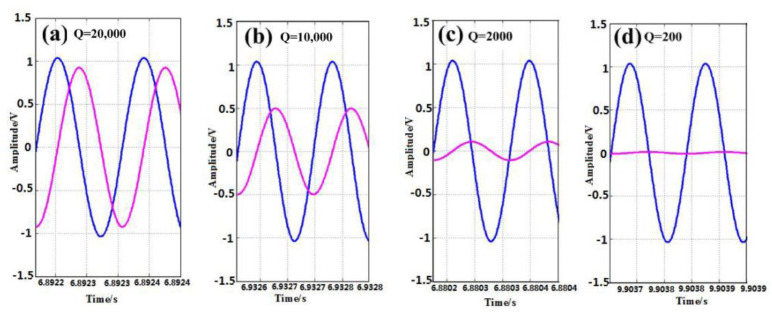
Output signals of the drive mode and detection mode with different Q.

**Figure 9:**
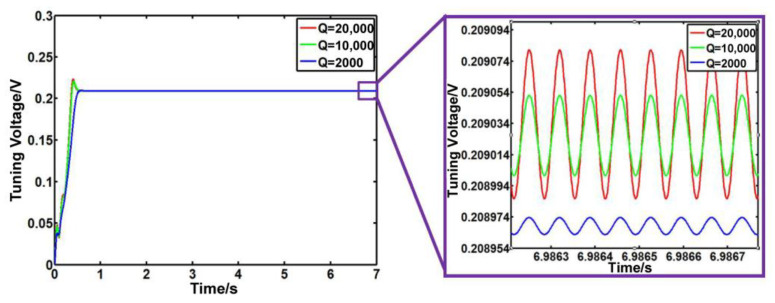
Output of the PI controller with different Q values.

In Equation (11), *Q* is the quality factor. We analyze the situations where *Q* is 200, 2000, 10,000, and 20,000. The output signals of the driving modal and detecting modal before modal matching are shown in Figure 8. As the quality factor decreases by order of magnitude, the detection signal decreases, although the phase difference between the driving signal and the detection signal is still maintained at 90°. When *Q* is 200, the detection signal is hardly amplified. Therefore, we analyze the accuracy of the system when *Q* is 2000, 10,000, and 20,000. From Figure 9, we find out that as *Q* increases, the fluctuation of the outputs of the PI controller increases, and the accuracy of the system reduces.

According to the simulation, it can be concluded that if the quality factor drops too much, it will affect the results of modal matching, and it will even affect the realization of modal matching. But when the magnitude is too high, the accuracy of the control system will decrease, which increases the difficulty of modal matching and causes a large error.

**Figure 10:**
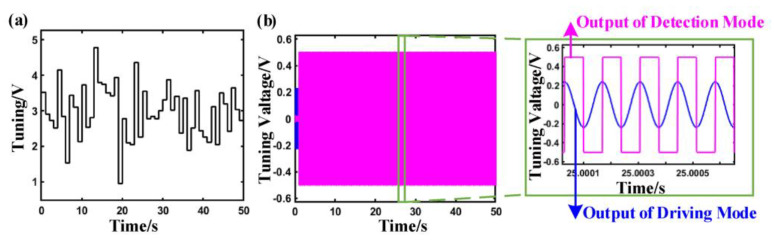
The input and output signal:(**a**) Input of angular velocity; (**b**) output signal of the control system with load when modal matching.

As shown in Figure 10a, the random angle signal is input into the system to explore the results of modal matching. The modal matching process after adding the angular velocity is shown in Figure 10b. It can be seen that the system response speed is fast after adding the angular velocity input. According to the stabilized enlarged signal, the gyro has achieved modal matching. The increase in the amplitude of the detected modal indicates that the detection mode is working.

## 5. Simulation of Fuzzy Controller for Mode Matching

**Figure 11:**
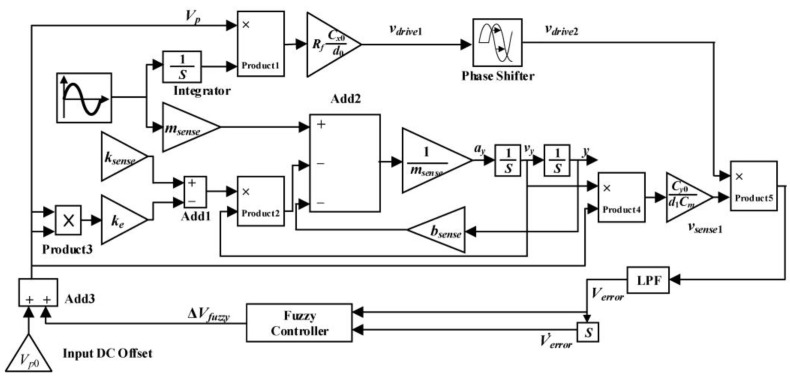
Simulink simulation block diagram of modal matching fuzzy control.

A fuzzy controller is utilized in the mode-matching system to improve the sensing performance. Then, the Simulink simulation block diagram of the simulation system with a fuzzy controller is shown in Figure 11. The fuzzy controller has the advantage of parameter adaptation, which can overcome the shortcomings that stem from processing errors and environmental drift.

The fuzzy controller for modal frequency matching adopts dual input and single output; the inputs are $V_{error}$ and ${\dot{V}}_{error}$, and the output ${∆V}_{fuzzy}$ of the controller is used to change the resonance frequency of the gyro detection mode. When fuzzing the membership function of $V_{error}$, we divide it into five fuzzy sets {NB, NS, ZO, PS, PB}, and set the scope of its universe to [−64, 64]. The membership function of the input ${\dot{V}}_{error}$ is divided into three fuzzy sets {N, Z, P}, and the scope of its universe is set to [−32, 32]. The membership function of the output ${∆V}_{fuzzy}$ is divided into five fuzzy sets {NB, NS, ZO, PS, PB}, and the scope of the universe is set to [−64, 64]. In the fuzzy controlling set, NB (Negative Big), NS (Negative Small), ZO (Zero), PS (Positive Small), and PB (Positive Big) are used to describe the five levels of parameter deviation. And fuzzy sets {N, Z, P} are used to describe the three levels of parameter deviation: N is Negative, Z is Zero, and P is Positive. 

According to the two inputs, the fuzzy rule library is formulated as shown in Table 1. When the deviation change increases in the negative direction and the deviation is also negative, we selected the maximum output in the positive direction, and vice versa. When the deviation increases in the negative direction and the deviation is not positive, we choose the maximum output in the positive direction and vice versa. When the deviation changes to 0 and the deviation increases slightly in the negative direction, the positive output is selected as a smaller output and vice versa. 

The quantification method of certainty is designed as a small method, and the defuzzification method adopts the weight method. We calculated the results according to the random point theory and used Table 2 for statistics.

**Figure 12:**
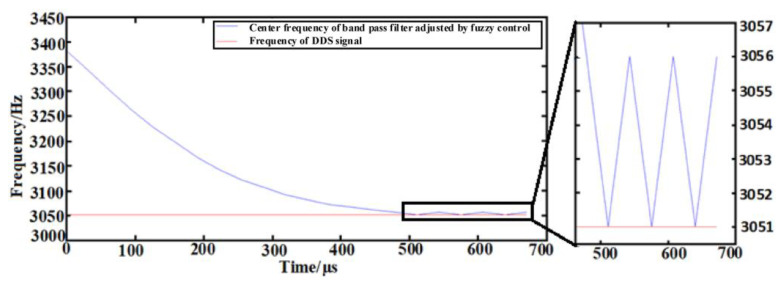
Center frequency and DDS frequency.

The initial value of the center frequency of the band-pass filter is 3381 Hz, and the sinusoidal signal frequency generated by the direct digital synthesizer (DDS) module is 3051 Hz. The address increments output by the fuzzy controller are sequentially searched to the center frequency of the band-pass filter corresponding to each address, which is shown in Figure 12. According to the partially amplified waveform, when reaching stability, the frequency of the band-pass filter changes in the range of 3051 Hz~3056 Hz, and the frequency error of the analog modal matching is within 5 Hz. And then, the frequency difference between driving and detecting is lower than the 1 Hz that was the base for the modal matching of micro-mechanical gyro. 

The simulation results show that the fuzzy PI controller can provide an excellent method for detection. The comparison of control performance with different controllers is shown in Table 3. The fuzzy PI displays a good method to decrease the response time, settling errors, and overshoots, which is the base for the stable detection of capacitive micromachined gyros. 

## 6. Conclusions

There are orthogonal errors in the detection mode when driving the gyro because of the process errors in the manufacturing of micro-mechanical gyro. In this paper, a PI closed-loop control system based on the phase relationship between the signals of driving mode and output orthogonal error is designed. The modal matching of the micro-mechanical gyro is realized through the changing of the input DC offset voltage. The frequency difference between the driving and detecting of the micro-mechanical gyro is controlled within 1 Hz using electrostatic control technology. The simulation model is built in Simulink to optimize the parameters of PI control, realizing the system to adjust the DC offset in 0.6 V and improving the response speed and stability of the system to achieve modal matching. The influence of system quality factor on the modal matching process is analyzed. A fuzzy controller is utilized in the system to ensure modal matching and improve sensing performance. The frequency difference during modal matching is controlled within 5 Hz, which has high control accuracy and stability. The feasibility of the system through the simulation input of angular velocity is verified. The results are helpful in improving the adaptability and accuracy of micro-mechanical gyros.

## Figures and Tables

**Table 1:** Fuzzy rule of modal matching controller.

${\dot{V}}_{error}$ $\text{\textbackslash{}}V_{error}$	NB	NS	ZO	PS	PB
N	PB	PB	ZO	NS	NS
Z	PB	PS	ZO	NS	NB
P	PS	PS	ZO	NB	NB

**Table 2:** The theoretical calculation results of the input and output of fuzzy controller.

$V_{error}$ $\text{\textbackslash{}}{\dot{V}}_{error}$	−35	−15	10	36
−66	64	48	47	32
−35	58	38	32	32
−20	32	22	18	20
10	−10	−12	−14	−13
38	−32	−32	−36	−54
68	−32	−41	−50	−64

**Table 3:** Comparison of control performance with difference controllers.

Method	Response Time	Settling Errors	Overshoot
PI controller	~1.5 s	~5%	~10%
Fuzzy PI controller	<0.4 s	<1%	<5%

## Data Availability

Not applicable.
